# Patterns of health-related quality of life and associated factors in Chinese patients undergoing haemodialysis

**DOI:** 10.1186/s12955-015-0308-3

**Published:** 2015-07-29

**Authors:** Eric Y. F. Wan, Julie Y. Chen, Edmond P. H. Choi, Carlos K. H. Wong, Anca K. C. Chan, Karina H. Y. Chan, Cindy L. K. Lam

**Affiliations:** Department of Family Medicine and Primary Care, The University of Hong Kong, 3/F Ap Lei Chau Clinic, 161 Main Street, Ap Lei Chau, Hong Kong; Institute of Medical and Health Sciences Education, The University of Hong Kong, 2/F William MW Mong Block, 21 Sassoon Road, Pokfulam, Hong Kong; School of Nursing, The University of Hong Kong, 4/F William MW Mong Block, 21 Sassoon Road, Pokfulam, Hong Kong

**Keywords:** Haemodialysis, Health related quality of life, SF-12v2, End stage renal disease, Chinese, Hong Kong

## Abstract

**Background:**

Haemodialysis (HD) is a life-saving but burdensome therapy for patients with end-stage renal disease (ESRD) which can have a detrimental impact on patients’ quality of life and outcomes. There is currently little data on the health related quality of life (HRQOL) of Chinese ESRD patients undergoing HD and this study sought to examine the patterns of HRQOL and its associated factors within this population, as well as in comparison with the general local population.

**Methods:**

A cross-sectional study of 244 ESRD patients receiving HD in the hospital and in the community in Hong Kong was conducted using the Short Form-12 Health Survey version 2 (SF-12v2). All study subjects were one-to-one matched with subjects in a Hong Kong general population database by sex and exact age. Independent t-tests were performed to compare the mean SF-12v2 scores between HD patients and the general population, followed by one-way analysis of variance with post hoc Tukey’s HSD tests to compare community-based haemodialysis, hospital-based haemodialysis and the general population. Multiple linear regressions were used to identify the factors (socio-demographic, clinical characteristics and comorbidities) associated with the HRQOL scores of ESRD patients receiving HD.

**Results:**

The SF-12v2 Physical Functioning, Role Physical, Bodily Pain, General Health and Physical Component Summary scores of HD patients were significantly lower than the age-sex adjusted general population. However, the SF-12v2 Mental Health and Mental Component Summary scores of HD patients were significantly higher than the corresponding general population. Poorer HRQOL was associated with being female, smoking, unemployment and hospital-based haemodialysis.

**Conclusions:**

HD patients had substantially poorer physical HRQOL but better mental HRQOL than the age-sex adjusted general population. Patients receiving HD in the community setting had better HRQOL. Reasons for these observations will need to be further investigated. Those patients who are female, smokers and unemployed may warrant more attention as their poorer HRQOL may be associated with poorer outcomes.

## Background

Chronic kidney disease (CKD) is an emerging global health problem. According to the Global Burden of Disease Study in 2010, chronic kidney disease rose from 27^th^ in 1990 to 18^th^ in 2010, in the list of causes of total number of global deaths [1]. CKD is a significant problem in Asia [2] with a prevalence of 11.9 % in Taiwan [3] and 10.8 % in Mainland China [4]. This is due to the increasing prevalence of key causes of CKD, hypertension and diabetes mellitus, which in turn increase the health burden attributable to CKD in the future [5].

Due to the progressive nature of CKD, many patients with CKD are likely to deteriorate to end-stage renal disease (ESRD) and eventually require some form of renal replacement therapy such as renal transplant, haemodialysis (HD) or peritoneal dialysis (PD). HD is the most effective mode of renal replacement therapy after renal transplantation, but is expensive and burdensome to the patient. In Asia, over 90 % of dialysis patients were on HD in China and Taiwan in 2005-2006 [6, 7] which contrasts sharply with the situation in Hong Kong where PD is designated as first-line therapy according to government policy. HD is reserved for those in whom PD has failed or who have medical contraindications towards PD [8, 9]. This accounts for the comparatively low percentage of HD patients in Hong Kong compared with the rest of the developed world. According to the most recent Hong Kong Renal Registry Report 2012, 1,246 (15.2 %) of patients in Hong Kong who needed renal replacement therapy were undergoing HD with the remainder on PD (43.6 %) or with a functioning renal transplant (40.3 %) [10]. Of the HD patients, over 60 % were treated in government-funded hospital-based renal units [10].

Though a life-saving therapy, HD can have a significant negative impact on the quality of patients’ lives. On a day-to-day basis, the frequency of dialysis and long duration of dialysis sessions limits patients’ independent living [11]. From a longer term perspective, HD can result in increased dependence on caregivers, loss of freedom, disturbance to family and to social life [12]. This, coupled with a stringent diet and fluid restriction, and the symptoms associated with ESRD itself, may be detrimental to overall health-related quality of life (HRQOL) [13–17].

HRQOL is an important health outcome for studies evaluating the impact of illness, clinical trials, audits of the quality of healthcare and analyses of cost-effectiveness [18, 19]. Furthermore, it has shown to be a clinically important dialysis outcome [20]. The majority of the published studies about the impact of HRQOL for ESRD patients receiving HD have been conducted in non-Chinese populations [13–17, 21]. However, HRQOL is a culture-specific construct as shown by the variation in relationship between HRQOL and outcomes such as patient survival, compliance and the patterns of medical practice [22–25] among international populations of ESRD patients. Awareness of culturally specific HRQOL issues in Chinese HD patients could facilitate a more patient-centred approach to health care.

However the comparison of HRQOL between HD patients in Hong Kong and HD patients may be affected by the aforementioned ‘PD first policy’ in Hong Kong. Patients undergoing HD in Hong Kong are likely to be more ill and to have poorer health status in contrast to the baseline state of HD patients in other countries where HD is the first choice for treatment of patients with ESRD and patients may be relatively healthier.

Understanding the impact of HRQOL of community-based and hospital-based HD patients with ESRD can provide a more comprehensive evaluation of quality of care than traditional clinical parameters alone. Knowledge of the difference in HRQOL between HD patients with ESRD and the general population can provide valuable insight on the physical and psychosocial burden of chronic illness and its treatment. Hence, the aim of this study was to evaluate the HRQOL of Chinese patients with ESRD undergoing HD. The specific objectives of the study were: (1) to compare HRQOL of community-based and hospital-based HD patients, (2) to compare HRQOL of HD patients with that of the Hong Kong general population; and (3) to identify the risk factors for poorer HRQOL in Chinese patients with HD.

## Methods

This was a cross-sectional observational study. Subjects were recruited from two different settings to include patients with a broad spectrum of characteristics. The subject recruitment period was from October 2012 to March 2013:

The community-based haemodialysis (CBHD) setting, for this study was the Haemodialysis Public Private Partnership (HD-PPP), a shared-care programme that gives eligible patients the option of receiving HD treatment in the community while continuing to be followed up in the renal clinic of the public hospital. Participants were predominantly PD patients who converted to HD for medical reasons (i.e. poor ultrafiltration, inadequate dialysis or frequent bacterial/fungal peritonitis on PD), who had good vascular access, were haemodynamically stable, and were mentally sound, ambulatory and independent for HD and who wished to join the CBHD programme. In this service provision model, HD services are purchased from non-government HD providers in the community without additional cost to the patient. In order to maintain the quality of care of the HD service, the protocols for HD were standardized across different community centres. During the study period, there were five community-based HD centres which provided HD services for 124 patients under the HD-PPP programme. All 124 HD-PPP patients were invited to join this study.

In a government-funded hospital-based HD (HBHD) setting, patients who were undergoing HD in thirteen government-funded hospitals across Hong Kong were recruited by convenience sampling. Eligible patients were invited by trained research assistants to take part in this study.

All subjects in community-based HD centres of the HD-PPP programme were recruited to join the study whilst patients in government-funded hospital-based HD Units were recruited by convenience sampling until a target sample size was reached. Subjects from both settings were excluded if they were aged < 18 years; could not understand Cantonese; had cognitive impairment, refused to participate; or were too ill to give consent (i.e. patients who reported they were too fatigued to complete the questionnaire or patients who were in isolation due to illness). Trained research assistants explained the nature of the study and invited subjects to participate. Those patients who agreed to participate and who provided written consent then completed a structured interviewer-administered questionnaire.

In order to compare the HRQOL of our study subjects against the Hong Kong general population, we identified a matching subject from the Hong Kong population study dataset for each subject in our study. This was possible because we had access to the original data set of the Chinese (Hong Kong) SF-12 Health Survey-Version 2 Hong Kong population study [26] (previously conducted by one of our co-authors), which surveyed 2533 subjects by random sampling. A match was defined as subjects of the same gender and exact age in year. This was done in accordance with well-accepted methodology to match for age and gender when comparing HRQOL to population norms. Age and gender were selected as matching criteria as they are strong factors affecting HRQOL whose effect must be controlled for when comparing to the general population. Previous studies also adopted this methodology to compare the HRQOL of patient populations against the general population [27, 28].

### Ethics approval

Ethics approval of this study was granted by all local Institutional Review Board (HKU/HA HKW: UW 10-366; HKEC: HKEC-2010-096; KEC/KCC: KC/KE-10-0208/ER-3; KWC: KW/EX/10-150 (34-17); CUHK/NTEC: CRE-2011.051); NTWC: NTWC/CREC/911/11). Informed consent was obtained from all patients.

### Outcome

The primary outcome in this study was HRQOL as measured by the Chinese (Hong Kong) Short Form-12 Health Survey version 2 (SF-12v2).

### Study instruments

#### The Chinese (Hong Kong) SF-12 Health Survey-Version 2 (SF-12v2)

The SF-12 v2 has been validated and normed on the general Chinese adult population in Hong Kong [26, 29]. It measures eight domains of HRQOL on Physical Functioning (PF), Role Physical (RP), Bodily Pain (BP), General Health (GH), Vitality (VT), Social Functioning (SF), Role Emotional (RE) and Mental Health (MH) on a scale with theoretical range from 0 to 100. A higher score indicates better HRQOL. The eight domain scores can be aggregated based on population-specific weights to calculate two summary scores, the Physical (PCS) and Mental Component Summary (MCS) scores.

#### Socio-demographic, clinical and comorbidity information

Demographic data including gender, age, marital status, educational level, smoking habit, and employment status were collected by a structured questionnaire. Clinical outcome measures such as dialysis efficacy as calculated by Kt/V and haemoglobin were extracted from medical records. The target value of Kt/V was defined as Kt/V ≥ 1.8 for patients receiving 2 HD sessions per week, or Kt/V ≥ 1.2 for patients receiving 3 HD sessions per week, or Kt/V ≥ 3.6 for weekly data [30]. The target haemoglobin was 9 g/dL or greater. Presence of co-morbidity was defined as having any of the following conditions: diabetes mellitus, hypertension, cancer, acute myocardial infarction, other chronic ischemic heart disease, congestive heart disease, stroke, peripheral vascular disease and chronic lung disease.

### Data analysis

Descriptive statistics were used to describe the characteristics and summarize the SF-12v2 scores of the subjects. Differences in baseline characteristics between CBHD and HBHD patients were tested by independent t-tests for continuous variables or chi-square tests for categorical variables. All HD patients were one-to-one matched with the Hong Kong general population by sex and exact age and independent t-tests were used to assess the differences in mean SF-12v2 scores [26]. Such comparisons of HRQOL scores were similarly performed in previous studies [27, 28]. The Cohen’s d effect sizes were also calculated and the value was interpreted as trivial for < 0.2, small for ≥ 0.2 and < 0.5, moderate for ≥ 0.5 and < 0.8 or large for ≥ 0.8 [31]. The differences in mean SF-12v2 scores across two settings and Hong Kong general population were further analyzed by one-way analysis of variance (ANOVA) with post hoc Tukey’s HSD test if there were significant differences between the three groups. The internal consistency of the SF-12v2 domain scores was assessed by Cronbach’s alpha coefficient, with a value ≥ 0.7 indicating adequate internal consistency [32]. The associations between risk factors (socio-demographics, clinical and comorbidity information) and HRQOL were assessed by multiple linear regressions.

All statistical analysis was performed using STATA Version 13.0 (StataCorp LP, College Station, Tex). All significance tests were two-tailed and findings with a p-value less than 0.05 were considered statistically significant.

## Results

### Subjects

A total of 305 eligible subjects (124 CBHD patients; 181 HBHD patients) were approached in five community-based HD centres and thirteen government-funded hospital-based HD Units. Amongst all, 244 patients consented to participate which gave a response rate of 80 % of all eligible patients approached. 110 (88.7 %) of 124 HD-PPP programme patients who were receiving HD in community-based centers completed the face-to-face interview. 134 (74.0 %) of 181 subjects who were receiving HD in government-funded hospital-based HD centers completed the face-to-face interviews.

The socio-demographic and clinical characteristics of CBHD and HBHD patients are displayed in Table 1. There were no statistical differences in socio-demographics except employment status and transportation means. More patients in HBHD were in active employment and travelled by taxi to their HD service. There was no significant difference between the two groups in dialysis adequacy (Kt/V) or hemoglobin level, two conventional clinical outcome measures. Moreover, both groups had similar proportions of patients suffering from co-morbidities.

**Table 1:** Socio-demographic and additional information between CBHD and HBHD patients

Factor	Total	CBHD patients	HBHD patients	P-value
	(n = 244)	(n = 110)	(n = 134)	
Socio-demographic				
Sex				0.757
Female	40.2 %	39.1 %	41.0 %	
Male	59.8 %	60.9 %	59.0 %	
Age (mean ± SD), year	56.6 ± 12.7	57.2 ± 13.0	56.0 ± 12.4	0.463
Educational level				0.696
No formal education	8.6 %	10.0 %	7.5 %	
Primary	27.0 %	23.6 %	29.9 %	
Secondary	53.7 %	55.5 %	52.2 %	
Tertiary	10.7 %	10.9 %	10.4 %	
Marital status				0.583
Unmarried	34.6 %	32.7 %	36.1 %	
Married	65.4 %	67.3 %	63.9 %	
Smoking status				0.152
Non-smoker	95.1 %	97.3 %	93.3 %	
Current smoker	4.9 %	2.7 %	6.7 %	
Working status				0.011*
Not working	79.1 %	86.4 %	73.1 %	
Working	20.9 %	13.6 %	26.9 %	
Come alone/Accompanied for HD session				0.494
Alone	83.6 %	81.8 %	85.1 %	
Accompanied by others	16.4 %	18.2 %	14.9 %	
Transportation to dialysis center				0.024*
Walk	8.2 %	8.2 %	8.2 %	
Public transport	74.6 %	81.8 %	68.7 %	
Taxi/ others	17.2 %	10.0 %	23.1 %	
Clinical and Co-morbidity information				
Kt/V				0.337
Target^a^ not achieved	26.8 %	29.6 %	23.8 %	
Target^a^ achieved	73.2 %	70.4 %	76.2 %	
Blood haemogloblin				0.239
<9 g/dL	22.8 %	19.4 %	26.1 %	
≥ 9 g/dL	77.2 %	80.6 %	73.9 %	
Co-morbidities				
Yes	78.1 %	77.8 %	78.4 %	0.914
No	21.9 %	22.2 %	21.6 %	

CBHD: Community-based haemodialysis; HBHD: Hospital-based haemodialysis; HD: Haemodialysis

*Significant with p-value < 0.05 by chi-square test or t-test as appropriate

^a^Target: Kt/V ≥ 1.8 for 2 HD sessions per week or Kt/V ≥ 1.2 for 3 HD sessions per week or Kt/V ≥ 3.6 for weekly data

### Health-related Quality of Life of Patients with HD

Table 2 presents the means and the standard deviations (SD) of HRQOL scores. Out of 2,831 subjects in the sex-age adjusted general population in Hong Kong, 233 were one-to-one matched with the HD patients under study by both sex and exact age and their SF-12v2 scores were compared. 11 out of 244 subjects could not be matched. Overall, the SF-12v2 PF, RP, BP, GH and PCS scores of HD patients were lower than the corresponding general population. It was unexpected to find that the SF-12v2 MH and MCS scores of HD patients were significantly higher than the corresponding general population. The results of Tukey’s HSD test showed that HBHD patients had poorer SF-12v2 PF, BP and PCS scores than the age-sex adjusted general population as well as the CBHD group. All the Cronbach’s alpha coefficients (Overall HD patients: 0.812; Age-sex adjusted Hong Kong general population: 0.842; CBHD patients: 0.823; HBHD patients: 0.809) of SF-12v2 domain scores exceeded the threshold of 0.7, implying the acceptable internal consistency reliability of the SF-12v2 scale.

**Table 2:** HRQOL scores of CBHD, HBHD patients and age-sex adjusted HK general population

SF-12v2	Overall HD patients^a^ (n = 233)	Age-sex adjusted HK general population^a^ (n = 233)	P-value	Effect size^b^	CBHD patients (n = 110)	HBHD patients (n = 134)	P-value for ANOVA	Significant difference***
	Mean (SD)	Mean (SD)			Mean (SD)	Mean (SD)		
Physical functioning	66.85 (32.55)	83.91 (26.03)	<0.001*	0.58	73.64 (29.68)	61.38 (34.09)	<0.001**	3 > 1 > 2
Role physical	71.35 (31.01)	79.08 (26.07)	0.004*	0.27	71.48 (32.27)	71.36 (30.11)	0.015**	3 > 2
Bodily pain	70.71 (31.62)	78.54 (25.97)	0.004*	0.27	77.50 (32.12)	64.93 (30.21)	<0.001**	1, 3 > 2
General health	37.55 (25.78)	46.93 (29.82)	<0.001*	0.34	41.36 (24.99)	35.00 (26.51)	<0.001**	3 > 2
Vitality	57.40 (24.15)	61.91 (29.09)	0.070	0.17	55.68 (24.10)	58.96 (24.02)	0.123	
Social functioning	77.25 (29.69)	81.97 (24.94)	0.064	0.17	78.18 (31.42)	77.43 (28.08)	0.242	
Role emotional	78.54 (25.42)	77.25 (23.01)	0.567	0.05	81.36 (25.10)	76.40 (24.94)	0.228	
Mental health	75.00 (19.97)	70.06 (18.92)	0.006*	0.25	75.34 (21.11)	74.81 (19.08)	0.020**	
Physical component summary score	42.77 (11.11)	47.50 (11.82)	<0.001*	0.41	45.71 (11.02)	40.26 (10.89)	<0.001**	1, 3 > 2
Mental component summary score	54.68 (9.14)	49.82 (10.41)	<0.001*	0.50	55.51 (7.96)	54.15 (9.94)	<0.001**	1, 2 > 3

HRQOL: Health-related Quality of Life; CBHD: Community-Based Haemodialysis; HBHD: Hospital-Based Haemodialysis; HK: Hong Kong

^a^Of 2,831 respondents in the study of population norm of the SF-12v2 in Hong Kong, 233 HD patients were matched to 233 general population subjects with sex and exact age. 11 subjects could not be matched

^b^Cohen’s d effect size between HD patients (overall) and HK general population

*Significant difference between HD patients (overall) and HK general population (P < 0.05)

**Significant difference between three groups (P < 0.05) by one-way ANOVA: Group 1, CBHD; Group 2, HBHD; Group 3, Hong Kong general population

***Significant difference between groups by post hoc Tukey’s HSD test (P < 0.05)

### Factors associated with HRQOL of ESRD patients receiving HD

Table 3 and Table 4 shows the factors associated with the domains of HRQOL, PCS and MCS scores using multiple linear regressions. CBHD patients had significantly higher scores in the SF-12v2 PF, BP, GH, RE and PCS scores than the HBHD patients. Being male was associated with higher SF-12 v2 PF, BP, RE and PCS scores. Being employed was associated with higher SF-12 v2 PF, RP, GH, VT, SF, RE and PCS scores. Smoking was associated with lower SF-12 v2 PCS but higher MCS scores. Compared with patients who went to HD sessions alone, those who were accompanied by others had poorer SF-12 v2 PF score. No factor was associated with the SF-12 v2 MH score.

**Table 3:** Factors associated with SF-12v2 at baseline by multiple linear regressions

	PF			RP			BP			GH			VT		
Independent variables	Coef.	95 % CI	P-value	Coef.	95 % CI	P-value	Coef.	95 % CI	P-value	Coef.	95 % CI	P-value	Coef.	95 % CI	P-value
Socio-demographics															
Male (vs. Female)	16.22	(7.26,25.17)	< 0.001*	8.00	(-1.98,17.98)	0.116	14.85	(4.91,24.79)	0.004*	3.30	(-4.71,11.32)	0.417	1.78	(-6.01,9.57)	0.653
Age	0.09	(-0.28,0.46)	0.624	0.05	(-0.37,0.46)	0.819	−0.07	(-0.48,0.35)	0.750	−0.08	(-0.41,0.25)	0.641	0.27	(-0.06,0.59)	0.105
Educational level (No formal education)															
Primary	2.66	(-14.07,19.38)	0.755	6.94	(-11.70,25.59)	0.464	−9.33	(-27.90,9.24)	0.323	8.47	(-6.51,23.44)	0.266	−1.23	(-15.79,13.32)	0.867
Secondary	−2.32	(-18.91,14.28)	0.783	−5.00	(-23.49,13.50)	0.595	−8.30	(-26.72,10.13)	0.375	1.76	(-13.09,16.62)	0.815	−3.50	(-17.93,10.94)	0.633
Tertiary	5.20	(-15.05,25.44)	0.613	−5.26	(-27.83,17.31)	0.646	−3.39	(-25.87,19.09)	0.766	−2.78	(-20.91,15.35)	0.763	−0.74	(-18.36,16.88)	0.934
Married (vs. Unmarried)	0.51	(-8.17,9.19)	0.908	4.57	(-5.10,14.25)	0.353	−3.47	(-13.11,6.17)	0.479	7.37	(-0.40,15.14)	0.063	2.91	(-4.65,10.46)	0.449
Smoker (vs. Non-smoker)	−12.80	(-31.27,5.66)	0.173	−3.70	(-24.29,16.88)	0.723	−7.79	(-28.29,12.72)	0.455	−4.50	(-21.03,12.03)	0.592	−1.99	(-18.06,14.08)	0.807
Working (vs. Not working)	17.49	(6.77,28.21)	0.002*	16.42	(4.47,28.37)	0.007*	3.54	(-8.36,15.44)	0.558	12.14	(2.54,21.73)	0.013*	11.84	(2.51,21.17)	0.013*
CBHD, Yes (vs. No)	11.31	(3.22,19.40)	0.006*	2.73	(-6.28,11.75)	0.551	12.33	(3.35,21.31)	0.007*	7.59	(0.35,14.83)	0.040*	−2.52	(-9.55,4.52)	0.481
Come alone/ accompanied for HD session, Accompanied by others (vs. Alone)	−21.26	(-33.76,-8.76)	0.001*	−2.16	(-16.09,11.78)	0.760	7.84	(-6.04,21.72)	0.267	−2.64	(-13.83,8.55)	0.642	−4.27	(-15.15,6.61)	0.440
Transportation to dialysis center (vs. Walk)															
Public transport	−3.18	(-18.10,11.74)	0.675	−2.34	(-18.97,14.29)	0.782	12.09	(-4.48,28.66)	0.152	−6.73	(-20.08,6.63)	0.322	−0.43	(-13.41,12.56)	0.949
Taxi/ others	−4.75	(-22.20,12.71)	0.592	−3.25	(-22.71,16.21)	0.742	10.26	(-9.12,29.64)	0.298	−10.22	(-25.85,5.41)	0.199	2.63	(-12.56,17.82)	0.733
Clinical and Co-morbidity information															
Kt/V,Target^a^ achieved (vs. Target^a^ not achieved)	−6.85	(-15.72,2.02)	0.129	1.11	(-8.77,10.99)	0.825	−1.37	(-11.22,8.47)	0.784	0.83	(-7.10,8.77)	0.836	−0.72	(-8.44,6.99)	0.854
Blood haemogloblin, ≥ 9 g/dL (vs. < 9 g/dL)	−0.91	(-10.34,8.51)	0.848	1.17	(-9.34,11.68)	0.826	−3.24	(-13.71,7.23)	0.542	−3.69	(-12.13,4.75)	0.390	1.01	(-7.19,9.22)	0.808
Comorbidities, Yes (vs. No)	−5.05	(-14.65,4.55)	0.301	−3.80	(-14.50,6.90)	0.485	4.06	(-6.60,14.72)	0.454	1.38	(-7.21,9.97)	0.752	−3.77	(-12.12,4.58)	0.375
Constant	60.62	(27.13,94.11)	< 0.001*	61.42	(24.09,98.75)	0.001*	56.80	(19.62,93.99)	0.003*	34.70	(4.72,64.68)	0.024*	43.54	(14.41,72.68)	0.004*

CBHD: Community-Based Haemodialysis; HD: Haemodialysis; PF: Physical Functioning; RP: Role Physical; BP: Bodily Pain; GH: General Health; VT: Vitality; CI: Confidence Interval

*Significant with p-value < 0.05

^a^Target: Kt/V ≥ 1.8 for 2 HD sessions per week or Kt/V ≥ 1.2 for 3 HD sessions per week or Kt/V ≥ 3.6 for weekly data

**Table 4:** Factors associated with SF-12v2 at baseline by multiple linear regressions

	SF			RE			MH			PCS			MCS		
Independent variables	Coef.	95 % CI	P-value	Coef.	95 % CI	P-value	Coef.	95 % CI	P-value	Coef.	95 % CI	P-value	Coef.	95 % CI	P-value
Socio-demographics															
Male (vs. Female)	4.70	(-4.69,14.08)	0.325	9.93	(2.00,17.85)	0.014*	−1.48	(-7.94,4.97)	0.651	7.55	(4.40,10.70)	< 0.001*	−0.17	(-3.06,2.73)	0.910
Age	−0.11	(-0.50,0.28)	0.587	−0.11	(-0.44,0.22)	0.508	−0.09	(-0.35,0.18)	0.527	−0.04	(-0.17,0.09)	0.517	−0.07	(-0.19,0.05)	0.240
Educational level (No fomal education)															
Primary	0.17	(-17.37,17.70)	0.985	−4.67	(-19.48,10.13)	0.534	3.84	(-8.22,15.89)	0.531	−0.63	(-6.52,5.25)	0.832	0.77	(-4.63,6.18)	0.778
Secondary	−8.66	(-26.05,8.74)	0.328	−12.90	(-27.59,1.78)	0.085	2.69	(-9.27,14.65)	0.658	−1.82	(-7.66,4.02)	0.539	−2.03	(-7.40,3.33)	0.455
Tertiary	−5.60	(-26.83,15.63)	0.603	−6.50	(-24.42,11.43)	0.475	6.28	(-8.32,20.87)	0.397	−2.46	(-9.59,4.66)	0.496	1.18	(-5.37,7.72)	0.724
Married (vs. Unmarried)	1.47	(-7.63,10.57)	0.751	2.25	(-5.44,9.93)	0.565	−1.71	(-7.97,4.54)	0.590	1.87	(-1.19,4.92)	0.229	1.34	(-1.46,4.15)	0.347
Smoker (vs. Non-smoker)	15.42	(-3.95,34.78)	0.118	4.72	(-11.62,21.07)	0.569	10.22	(-3.09,23.53)	0.132	−7.27	(-13.76,-0.77)	0.029*	6.64	(0.67,12.62)	0.029*
Working (vs. Not working)	11.30	(0.06,22.54)	0.049*	11.17	(1.68,20.66)	0.021*	1.84	(-5.88,9.57)	0.638	4.60	(0.83,8.37)	0.017*	2.15	(-1.32,5.61)	0.223
CBHD, Yes (vs. No)	2.71	(-5.77,11.19)	0.530	8.54	(1.39,15.70)	0.020*	1.62	(-4.21,7.44)	0.585	4.77	(1.93,7.62)	0.001*	2.46	(-0.15,5.08)	0.065
Come alone/ accompanied for HD session, Accompanied by others (vs. Alone)	−5.64	(-18.74,7.47)	0.397	−1.79	(-12.85,9.28)	0.750	−2.89	(-11.90,6.12)	0.528	−3.34	(-7.74,1.05)	0.135	0.98	(-3.06,5.02)	0.633
Transportation to dialysis center (vs. Walk)															
Public transport	−13.18	(-28.82,2.47)	0.098	3.50	(-9.71,16.71)	0.602	−2.30	(-13.06,8.46)	0.674	0.66	(-4.59,5.91)	0.804	−0.21	(-5.03,4.61)	0.931
Taxi/ others	−11.47	(-29.77,6.83)	0.218	8.25	(-7.20,23.70)	0.294	−2.80	(-15.38,9.79)	0.661	−1.39	(-7.53,4.75)	0.655	0.09	(-5.56,5.73)	0.976
Clinical and Co-morbidity information															
Kt/V,Target^a^ achieved (vs. Target^a^ not achieved)	−3.77	(-13.06,5.53)	0.425	1.18	(-6.67,9.02)	0.768	−4.33	(-10.72,2.06)	0.183	−0.42	(-3.54,2.69)	0.789	−0.43	(-3.30,2.43)	0.766
Blood haemogloblin, ≥ 9 g/dL (vs. < 9 g/dL)	−5.06	(-14.94,4.83)	0.314	−2.74	(-11.08,5.61)	0.518	−0.80	(-7.60,5.99)	0.816	0.86	(-2.46,4.18)	0.609	−0.09	(-3.14,2.96)	0.954
Comorbidities, Yes (vs. No)	2.32	(-7.74,12.39)	0.649	−3.51	(-12.00,4.99)	0.416	−0.15	(-7.07,6.77)	0.965	−0.30	(-3.68,3.07)	0.860	1.91	(-1.19,5.02)	0.225
Constant	98.58	(63.47,133.69)	< 0.001*	79.74	(50.10,109.39)	< 0.001*	83.43	(59.29,107.57)	< 0.001*	38.35	(26.57,50.13)	< 0.001*	55.58	(44.75,66.41)	< 0.001*

CBHD: Community-Based Haemodialysis; HD: Haemodialysis; SF: Social Functioning; RE: Role Emotional; MH: Mental Health; PCS: Physical Component Summary; MCS: Mental Component Summary; CI: Confidence Interval

*Significant with p-value < 0.05

^a^Target: Kt/V ≥ 1.8 for 2 HD sessions per week or Kt/V ≥ 1.2 for 3 HD sessions per week or Kt/V ≥ 3.6 for weekly data

## Discussion

In relation to the impact of HD on HRQOL as measured by the SF-12v2, our HD patients had poorer HRQOL in the PF, GH domains and PCS with small to moderate effect sizes of 0.58 (PF), 0.34 (GH) and 0.41 (PCS) than the adjusted general population. This finding was consistent with the results of a previous study in which ESRD patients had poorer HRQOL as measured by the physical dimension of the Short Form-36 [33]. An American study also revealed that the SF-36 PF domain of ESRD patients was substantially lower (39.9) than the US general population [34]. A systematic review found that the weighted prevalence of the symptoms of fatigue and tiredness in ESRD patients was 71 % and 44 % of ESRD patients who suffered from sleep disturbance [35], which might have led to impairment in activities of daily living (RF) and of physical functioning (PF) and poor general health perception (GH). Moreover, many patients in Hong Kong are influenced by belief in the theories of traditional Chinese Medicine, in which weak kidney function is detrimental to overall health (GH). HD patients also had lower SF-12v2 BP score than the general population, which might be attributable to the easy muscle fatigue associated with ESRD.

It was encouraging, although unexpected, that HD patients had better SF-12v2 MH and MCS scores than the age-sex matched general population, with small to moderate effect sizes of 0.25 (MH) and 0.5 (MCS). Cross-sectional studies in western populations reported that the mental health in HD patients was not different from that of the general population or from patients who had received renal transplants [36–40]. A study in the US suggested that there was minimal negative impact of ESRD on the MH domain of the SF-36 (5 on a 100-point scale lower than the general population). Previous studies found that HD patients with psychological adaptation had greater appreciation of life and better mental health [41, 42] which might be the explanation for the high perceived mental-health related quality of life among our Chinese HD patients. As HD patients need to attend HD 2 or 3 times a week for several hours each time, there may be opportunity for the nursing staff to build up rapport, enhance knowledge, enable coping and provide more psychological support to HD patients that are beneficial to mental health. Another possible explanation may be found in the “response shift theory” [43]. ESRD patients might reframe their expectation, and their conceptualization of health-related quality of life over the course of the disease trajectory, which in turn leads to “better” MH and MCS scores than the general population. Further qualitative studies would be useful to explore the illness experience, coping and mental health of ESRD and HD patients.

Of interest, both mental or physical health-related summary scores of our Chinese HD patients in Hong Kong were higher than those of HD patients in other countries including Singapore [13], Japan [44], Brazil [45], Europe [44], the United Kingdom [46] and the US [44, 47, 48]. Our patients also had better physical and mental component summary of HRQOL (PCS: 42.8; MCS: 54.7) than others of the same ethnicity, namely HD patients in mainland China (PCS: 36.2; MCS: 43.8) and Taiwan (PCS: 36.3; MCS: 37.5) [49, 50]. The results revealed that the perceived life impact of disease is culture-specific but is also influenced by health beliefs as well as the country’s health care system. Health care financing may also influence HRQOL especially for people with serious chronic diseases that require expensive life-saving treatments such as HD [44]. For example, HD patients in the US had better SF-36 MCS than those in Japan and Europe, but HD patients in Japan had better SF-36 PCS of than those in the US and Europe [44]. Interpretation of HRQOL findings must take into consideration the cultural and health care context of the population.

Compared with patients undergoing CBHD, those patients undergoing HBHD had poorer SF-12v2 PCS, PF and BP scores, but no significant difference in the SF-12v2 MCS or other domain scores, by ANOVA and after adjustment of confounders by multiple regressions. This was different from the findings in the literature which showed no difference between groups in both physical and mental components [51]. This may be due to the “self-selection” of more physically fit patients for CBHD. Although the socio-demographics and proportion of presence of co-morbidities of the two groups were statistically similar, the severity level of comorbidities in HBHD group may be higher than that in CBHD because of the requirement that patients be independent and ambulatory for the CBHD group. More detailed exploration for any difference in the health status of the HBHD and CBHD patients on entry into programme and a longitudinal study on the change in HRQOL of both groups would provide insight into whether HD setting has an independent effect on HRQOL. On the other hand, there were some patients in the hospital, who met the referral criteria, but refused to transfer to the community centre because they were worried about transferring to an unfamiliar environment.

The results of socio-demographic factors associated with the HRQOL of HD patients showed that male HD patients had better scores in physical domains of HRQOL but no difference in mental domains compared with female patients. Numerous studies had reported poorer HRQOL in several or all dimensions in female compared with male dialysis patients [50, 52–54]. The finding that females have poorer HRQOL is consistent with that found in the general populations [50].

We found that employed HD patients had better SF-12v2 scores in several HRQOL domains including PF, RP, GH, VT, SF, RE as well as the PCS which is consistent with findings cited in the literature [33, 50, 55, 56]. Patients are less likely to perceive impairment in their activities of daily living when they are able to continue active employment, but the low employment rate is notable in our HD population. Other studies found that the need to frequently attend HD sessions or disability discrimination [57, 58] to be reasons for the low employment rate. Nonetheless, counselling and vocational rehabilitation should be provided to encourage dialysis patients who are able to work to do so, even on a voluntary basis, for enhancement of HRQOL.

It is well known that smoking is a risk factor for increasing disease burden [59] which may explain the inverse relationship observed between smoking and SF-12v2 PCS, but paradoxically a direct relationship was found between smoking and SF-12v2 MCS. The benefit to mental health from smoking may be from the pleasure or relaxation derived from the habit [60], though this is inconclusive due to the cross-sectional design and small proportion of smokers in our study (<5 %).

HD patients who came to HD centre accompanied by others had much lower HRQOL in PF than those who attended alone. This is expected, as patients with poorer physical functioning were might not have been able to attend HD session alone and the accompanying person was usually a domestic helper whose responsibility was to physically assist the patient to and from the HD session. Conversely, patients who were physically fit should have had no problem to attend HD session alone.

We found that clinical measures and the presence of co-morbidities were not associated with HRQOL of HD patients. Although dialysis adequacy and anaemia are key dialysis outcome measures and predictors of morbidity and mortality [61–63], they were not associated with patients’ HRQOL. Similarly the presence of co-morbidities was not associated with HRQOL. Some studies observed that there was a positive correlation between haemoglobin level and perceived HRQOL [64–66] and a negative correction between co-morbidity and HRQOL [50, 67–69]. However, other reports were consistent with our results, showing that the Kt/V and haemoglobin did not significantly correlate with HRQOL of dialysis patients [70, 71].

### Strengths and limitations of this study

There were two main strengths of the present study. First, we were able to determine the impact of ESRD and HD on HRQOL through normative comparisons of HRQOL between our patients and age-sex matched subjects from the general population. Second, HD patients were recruited in both hospital and community settings which permitted the exploration of possible HRQOL differences between patients undergoing dialysis in contrasting settings.

There were a number of limitations to this study. Firstly, all subjects were recruited from the community centres and government-based hospitals, and the results may not be generalizable to ESRD patients undergoing dialysis in the private settings. Second, the HRQOL was measured by the SF-12v2. As a generic measure, the SF-12v2 might not be sufficiently sensitive as disease-specific measures to detect differences between HD patient groups. For example, the finding that no factor was associated with mental health domain of the SF-12v2, or the lack of association between clinical parameters and HRQOL scores might be due to the insensitivity of the measure. Further study with the dual use of generic and disease-specific measures may help evaluate HRQOL of HD patients more comprehensively. Third, additional clinical parameters such as albumin, creatinine and calcium would help to obtain a more complete picture of which dialysis outcome measures relate to HRQOL of HD patients. Fourth, it would have been be worthwhile to investigate the difference in HRQOL between two-times and three-times weekly HD, had the data been available. Lastly, as a cross-sectional study, only preliminary information about association of HRQOL and risk factors was possible to be obtained and longitudinal studies are needed to evaluate any causal relationship as well as the change of HRQOL among HD patients in different settings.

## Conclusions

Different from most other previous studies, our study demonstrated HD patients as having better mental health-related quality of life when compared to the general population. CBHD patients seemed to perceive better HRQOL in physical domains but were no different in mental domain scores from HBHD. Factors associated with poorer HRQOL included female gender, smoking, and not working. Features of the HD setting as well as other modifiable factors should be further investigated to improve HRQOL for HD patients. Longitudinal studies would help establish causation and evaluate changes in HRQOL over time.

## Abbreviations

ANOVA: Analysis of variance
BP: Bodily pain
CBHD: Community-based haemodialysis
CKD: Chronic kidney disease
ESRD: End stage renal disease haemodialysis
GH: General health
HBHD: Hospital-based haemodialysis
HD: Haemodialysis
HD-PPP: Haemodialysis public private partnership
HRQOL: Health related quality of life
MCS: Mental component summary
MH: Mental health
PCS: Physical component summary
PD: Peritoneal dialysis
PF: Physical functioning
RE: Role emotional
RP: Role physical
SF: Social functioning
SF-12 v2: The Short Form-12 Health Survey version 2.
VT: Vitality
